# Tree cover in Central Africa: determinants and sensitivity under contrasted scenarios of global change

**DOI:** 10.1038/srep41393

**Published:** 2017-01-30

**Authors:** Julie C. Aleman, Olivier Blarquez, Sylvie Gourlet-Fleury, Laurent Bremond, Charly Favier

**Affiliations:** 1 Ecology and Evolutionary Biology, Yale University, New Haven, USA; 2 Institut des Sciences de l’Evolution de Montpellier, Université de Montpellier, CNRS, IRD, EPHE, Montpellier, France; 3 UR Biens et services des écosystèmes forestiers tropicaux (CIRAD), Montpellier, France; 4 Département de Géographie, Université de Montréal, Montréal, Québec, Canada

## Abstract

Tree cover is a key variable for ecosystem functioning, and is widely used to study tropical ecosystems. But its determinants and their relative importance are still a matter of debate, especially because most regional and global analyses have not considered the influence of agricultural practices. More information is urgently needed regarding how human practices influence vegetation structure. Here we focused in Central Africa, a region still subjected to traditional agricultural practices with a clear vegetation gradient. Using remote sensing data and global databases, we calibrated a Random Forest model to correlatively link tree cover with climatic, edaphic, fire and agricultural practices data. We showed that annual rainfall and accumulated water deficit were the main drivers of the distribution of tree cover and vegetation classes (defined by the modes of tree cover density), but agricultural practices, especially pastoralism, were also important in determining tree cover. We simulated future tree cover with our model using different scenarios of climate and land-use (agriculture and population) changes. Our simulations suggest that tree cover may respond differently regarding the type of scenarios, but land-use change was an important driver of vegetation change even able to counterbalance the effect of climate change in Central Africa.

Forest and savanna constitute the two main ecosystems in Central Africa. Forests are characterized by a closed canopy with mostly non-pioneer light demanding species in the overstory and shade-tolerant species in the understory, while savannas are defined by a continuous layer of C_4_ grasses with a varying density of disturbance-tolerant woody species^1^. Thus, tree cover constitutes a key variable that differentiates forest and savanna, and which reflects variation in vegetation structure within the savanna biome^2^. Tree cover also characterizes landscape structure and functionality, especially with effects on carbon storage, albedo, and biodiversity. Hence, tree cover is often used to characterize tropical biomes at broad scales^2^,^3^,^4^,^5^,^6^.

Climate is considered as the primary driver of tree cover in Africa^5^, where rainfall constrains maximum tree cover and where disturbances, especially fire and herbivores^7^,^8^,^9^,^10^, reduce tree cover from its maximum in a less predictable way^2^. Soil variables are also important in determining tree cover because they impact fertility and water availability^11^. Therefore, tree cover in savanna is the result of complex interactions between climate, soil characteristics and disturbance regimes^11^,^12^. The relative importance of each of these variables in determining tree cover is still a matter of some debate^11^, and vary regarding the location, spatial scale and extent of analyses^2^,^3^,^5^,^13^. Moreover, anthropogenic activities are rarely taken into account in analyses of tree cover, but human land-use has been shown to be a strong predictor of tree cover at the continental scale^14^. The effects of land-use change are not necessarily straightforward; while increasing cropland area obviously reduces tree cover^15^, pastoralism may have an opposite effect on vegetation structure, as overgrazing and changes in fire regime have been shown to be responsible for woody encroachment^16^,^17^.

In the current context of climate change and modifications of land-use practices, more local and regional studies are needed in order to understand the interactions between climatic variables, disturbance regimes and agricultural practices. Indeed, future climate in Africa is expected to change severely, especially rainfall patterns and distribution^18^. These changes are likely to influence vegetation characteristics, ecosystem biodiversity and, at larger scale, global biome distribution^19^,^20^. Climate change, however, is not the only threat to African forest and savanna. Because local populations directly rely on them for their livelihoods^21^, they sustain increasing human impacts. Agricultural lands are expanding as populations grow rapidly in Africa^22^, while savanna areas are increasingly targeted for biofuel and intensive crops production^23^,^24^. Land-use changes and intensification result in forest and savanna degradation, fragmentation and biodiversity loss^25^,^26^ and can be critical for the future of ecosystems, directly threatening the sustainability of ecosystem services provisioning. Some modelling effort has been recently done for taking into account scenarios of land-use change in Africa^14^,^25^,^27^, highlighting the importance of agricultural practices and mitigation policies in the future of African ecosystems^14^. While Heubes *et al*.^27^ focused on West Africa where human impact is high and has already strongly modified the vegetation^28^, Aleman *et al*.^29^ performed their analyses in all sub-Saharan Africa, potentially masking regional peculiarities in agricultural practices and directions in management policies. We thus emphasize that local to regional-scale studies can also be crucial for understanding drivers of vegetation structure and improving the projections under scenarios of future changes.

Here we focused on an area centred in Central African Republic, which shows a clear vegetation gradient from tropical forest to open grasslands. This region has been subjected to violent political conflicts for decades and is facing a lack of transport infrastructure. The combination of political instability, isolation and particularly low population densities^30^ has prevented up to now intensive agricultural conversion^31^ and favoured the maintenance of rather traditional agricultural and land-use practices^32^. Focusing on this region is thus markedly important. From a theoretical point of view, it offers the opportunity to study how climate and traditional practices have determined savanna extent and tree cover. From an environmental policy point of view, when the region eventually stabilizes, there will be a surge of land conversion: this region is, for instance, part of the areas targeted for future biofuel production and agriculture intensification^23^,^24^. Then, scientific guides for land-use management and conservation design will be desperately needed.

The objective of this paper is thus to examine the roles of climate, edaphic variables, fire and agricultural practices (here cropland and pasture densities) in determining tree cover, and to use projections from the Coupled Model Intercomparison Project 5 (CMIP5)^33^ to generate correlative predictions about future tree cover. The frequency distribution of tree cover data derived from Vegetation Continuous Field product of the MODerate-resolution Imaging Spectroradiometer (MODIS) sensor^34^ is multimodal in our study area^4^, and is used to define four vegetation classes: three classes of savanna with increasing tree cover, and one forest class. The four modes recorded in this study area are not apparent in continental analyses^3^,^14^, probably because large-scale agricultural conversion mask the specificity of local-scale practices from which different vegetation structures emerge^13^. We therefore calibrated a Random Forest model of tree cover using remote sensing data and large databases of climate, anthropogenic and edaphic data. Random Forest models have been shown to accurately reproduce tree cover patterns at the continental scale in Africa^14^, and to be appropriate for use in prediction^35^. This model calibrated locally was able to pick up more information about current agricultural practices on tree cover in Central Africa than previous continental-scale study^14^. We used this model to quantify tree cover changes and vegetation classes’ shifts in response to highly contrasted scenarios of climate and agricultural practices changes as defined by the underlying emission and socio-economic scenarios of the different Representative Concentration Pathways^36^.

## Results

### Current determinants of tree cover and savanna structural classes

**Figure 1:**
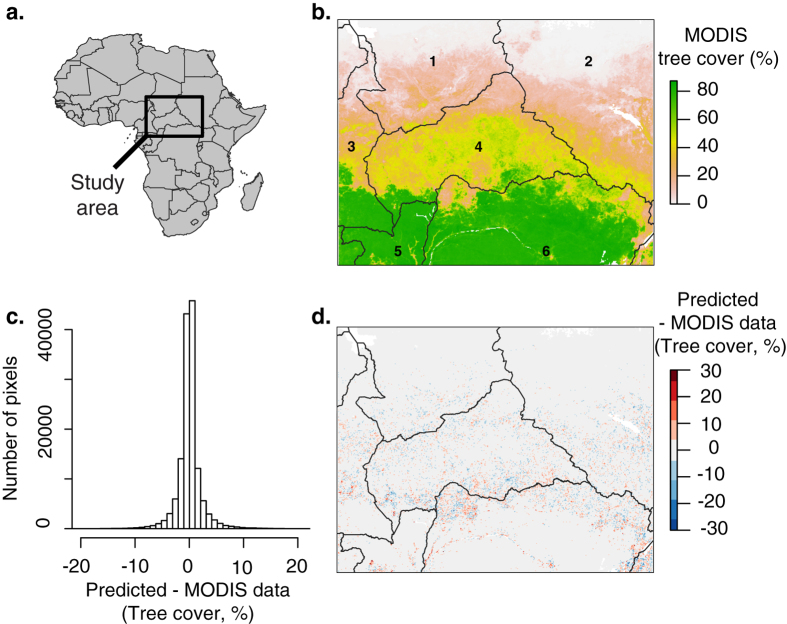
Location of the study area (**a**) and spatial distribution of MODIS tree cover data (**b**), with 1: Chad, 2: South Sudan, 3: Cameroon, 4: Central African Republic, 5: the Republic of Congo, and 6: the Democratic Republic of Congo. Histogram (**c**) and spatial distribution (**d**) of the difference between predicted tree cover using our Random Forest model and MODIS tree cover. This also represents the map of the opposite of the model’s residuals. The maps were generated using R version 3.1.3^85^.

**Figure 2:**
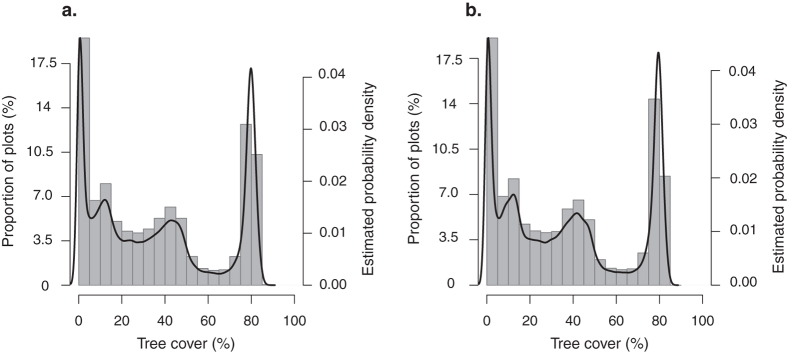
Histograms of tree cover for MODIS data (**a**) and predicted using our model (**b**). The black curves represent the estimated probability density of plots.

The Random Forest model predicted tree cover with very high accuracy (Fig. S1, R^2^ = 0.97, *P*-value < 0.01). Moreover, the residuals of the model were not spatially correlated (Moran’s I = 0.003, *P*-value = 0.10), such that the spatial pattern of tree cover in our study area was well captured (Fig. 1d) and the differences between predicted tree cover and MODIS data were very low (Fig. 1c,d). In our study area, MODIS tree cover is multimodal and clearly highlights four modes (Fig. 2a); the model captured this multimodality with high precision too (Fig. 2b). We thus defined four vegetation classes based on these four modes; these vegetation classes were also very accurately modelled with only 4.0% of misclassified pixels.

**Figure 3:**
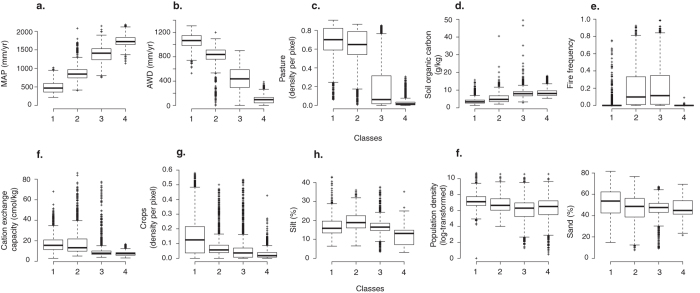
Boxplots of the ten predictors for each of the vegetation class computed in a subset points of the calibration dataset (N = 10,000).

The first class, characterized in our study by a TC ≤ 5%, i.e. almost no tree detected, corresponds to grassland savannas and bushlands (hereafter *grassland/bushland*) of the Sahelian biogeographic area^37^, where the accumulated water deficit (AWD) is the highest (Fig. 3b). Within this class we cannot distinguish between grassland and shrubland, as the MODIS tree-cover dataset underestimates woody cover smaller than 5-m in height^34^. The second class is characterized by 5% < TC ≤ 28%. Here called *sparsely wooded savannas*, this class corresponds to a vegetation characteristic of the Sahelo-Sudanian zone in the Central African Republic phytogeographic classification^38^ and to a class of wooded savanna in the description of the vegetation type in Chad^39^, where AWD is intermediate (Fig. 3). The *grassland/bushland* class and the *sparsely wooded savannas* class feature the greatest extent of pasture (Fig. 3). For the third class, tree cover is 28% < TC ≤ 65%, and the vegetation corresponds to *wooded and woodland savannas* (mosaics of savanna and forest of the Sudanian biogeographic area^37^). These two vegetation classes (*sparsely wooded savannas* and *wooded and woodland savannas*) exhibit the highest fire activity (Fig. 3e). Finally, the fourth class corresponds to *forest* (TC > 65%) and is chiefly located in areas with the lowest AWD and the highest annual precipitation (Fig. 3a). The soil organic carbon concentration is the highest for the *woodland savanna* and *forest* classes (Fig. 3d).

**Figure 4:**
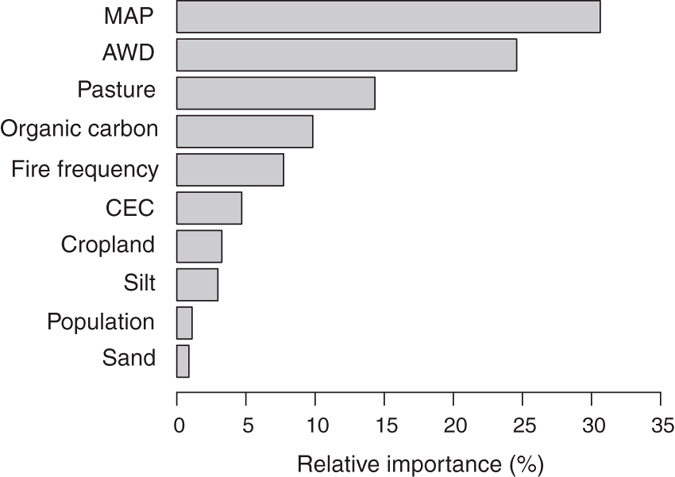
Relative importance values of the different model predictors in determining tree cover. See Table 1 for descriptions of the variables.

The influence of each of the predictors was quantified by computing their relative importance in the model (Fig. 4) and their partial dependence on tree cover (Fig. S2). Mean annual precipitation (MAP) and accumulated water deficit (AWD) were the two most important variables in our model; tree cover increased with increasing MAP and decreasing AWD (Figs 4 and S2). Anthropogenic variables were also important, especially the density of pasture per pixel, followed by fire frequency, cropland and population densities (Fig. 4). Not surprisingly, all anthropogenic variables tended to decrease tree cover (Fig. S2). As pasture density increased up to 0.8, tree cover decreased. Moreover, for null fire frequency, tree cover was the highest and monotonically decreased for increasing fire (Fig. S2). Cropland and population density had a smaller, but still negative, impact on tree cover. Lastly, edaphic variables were less important, except for the soil organic carbon concentration, which constitutes the fourth most important variable (Fig. 4) and may reflect vegetation impacts on soil, and not vice-versa. Tree cover showed a strong positive dependence on organic carbon up to 10 g/kg, then a negative one until 15 g/kg. Beyond this threshold, no dependence was recorded (Fig. S2).

### Future projections

**Figure 5:**
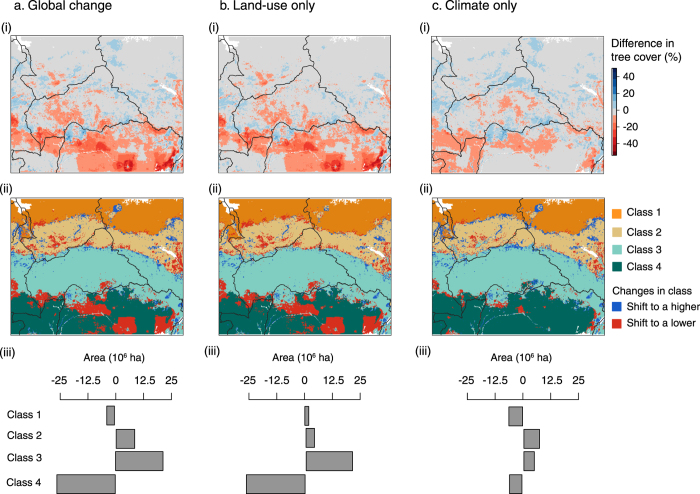
Changes in tree cover and classes’ spatial distribution for RCP 2.6. The first panel represents differences in tree cover between simulated future and current tree cover values (i), the second panel represents the spatial distribution of the four vegetation classes and their shifts for 2070 (ii), and the third panel represents the changes in area occupied by the four vegetation classes in 2070 (iii) for global change (**a**), land-use change only (**b**) and climate change only (**c**) scenarios. The maps were generated using R version 3.1.3^85^.

We used four scenarios (RCPs 2.6, 4.5, 6.0 and 8.5) of climate (MAP and AWD) and land-use (pasture, cropland and population densities) changes to simulate future tree cover in Central Africa, and two main trends seemed to emerge. First, for RCPs 2.6 and 8.5, respectively the most optimistic and pessimistic scenarios of emissions, tree cover changes and shifts in vegetation classes were surprisingly similar (Figs 5 and S4). For these two scenarios, the major driver of change was land-use modifications corresponding to increasing cropland areas for RCP 2.6 (Fig. S3b) and to increasing both cropland and pasture land for RCP 8.5 (Fig. S3a,b). Tree cover changes were greatest in the *forest* class, whose area decreased by more than 25 × 10^6^ ha for both scenarios (Figs 5b and S4b). For all scenarios, land-use changes were responsible for decreasing tree cover within all vegetation classes (Figs 5b and S4b). Shifts between classes were spatially structured and mainly clustered in areas where classes transitioned. The simulations for climate alone also predicted some tree cover changes, but these were somewhat milder compared to land-use change only (Fig. 5b,c), even for RCP 8.5 (Fig. S4b,c) where rainfall and water deficit changes are predicted to be the highest (Fig. S3c,d). Still, for RCP 2.6, tree cover decreased in the *forest* class as a result of increasing water deficit (Fig. S3d) and more than 10 × 10^6^ ha shifted to the *wooded and woodland savanna* class (Fig. 5c). Conversely, for this scenario, tree cover increased in the three savanna classes (Fig. 5c). This triggered an increase in the two intermediate savanna classes’ areas, *wooded and woodland savannas* and *sparsely wooded savannas,* but the *grassland/shrubland* class decreased because of the tree cover increase, due to increasing annual precipitation (Fig. 5c, Fig. S4c). Tree cover changes for RCP 8.5 followed a similar trend, but were more substantial and triggered more shifts between vegetation classes (Fig. S4c). Increasing MAP for this scenario led to an increase in tree cover in vegetation classes situated in North of Cameroon, Central African Republic (CAR) and south of Chad and South Sudan (Fig. S4c). Meanwhile, the increase in temperature, and thus AWD in southern parts of Cameroon, CAR and northern parts of Democratic Republic of Congo (DRC) resulted in tree cover decrease in *forest* areas (Fig. S4c).

**Figure 6:**
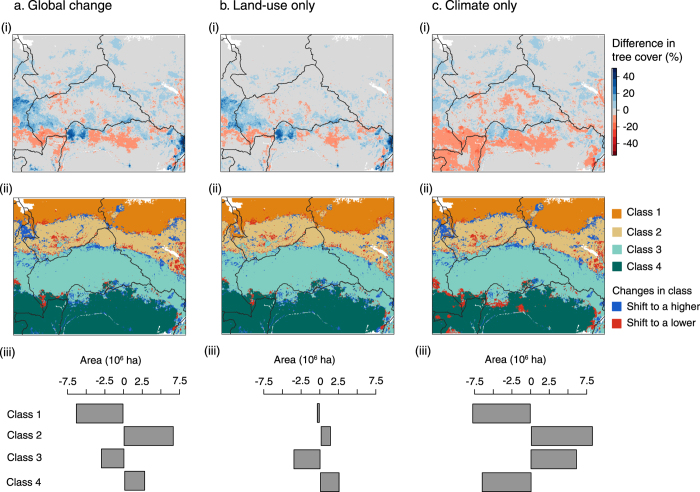
Changes in tree cover and classes’ spatial distribution for RCP 4.5. The first panel represents differences in tree cover between simulated future and current tree cover values (i), the second panel represents the spatial distribution of the four vegetation classes and their shifts for 2070 (ii), and the third panel represents the changes in area occupied by the four vegetation classes in 2070 (iii) for global change (**a**), land-use change only (**b**) and climate change only (**c**) scenarios. The maps were generated using R version 3.1.3^85^.

For the two other scenarios, RCPs 4.5 and 6.0, the main driver of classes’ shifts was climate (Figs 6 and S5). For RCP 4.5, however, tree cover changes due to land-use change were greater in magnitude but highly localized (Fig. 6b), while climate change effects were more spatially extensive (Fig. 6c). For this scenario, the net effect of climate and land-use changes was antagonistic. Increasing water deficit as a result of increasing temperature was responsible for decreasing tree cover in *forest*, with 7.5 × 10^6^ ha of this class shifting into to the *wooded and woodland savanna* class (Fig. 6c). However, for the land-use change-only scenario, tree cover increased in this savanna class, as a result of both reducing pasture and cropland in order to increase forest plantation^40^. The *forest* class increased in area, while the *wooded and woodland savanna* class decreased (Fig. 6c). Increasing MAP in Sahelian areas of Cameroon, Chad and South Sudan (Fig. 6c), increased tree cover and triggered shifts from *grassland/shrubland* to *sparsely wooded savanna*. Both climate and land-use change resulted in an increase in forest and *sparsely wooded savanna* area, while *wooded and woodland savanna* and *grassland/shrubland* decreased (Fig. 6a). Results for RCP 6.0 gave similar results (Fig. S5a).

## Discussion

In this study, we used a Random Forest algorithm to correlatively model tree cover using climatic, edaphic and anthropogenic data in Central Africa. This type of model has previously been used to model tree cover in all sub-Saharan Africa^14^ and to model biome shifts in the Indian subcontinent with high accuracy^41^. The model obtained here also predicted tree cover very accurately, without spatial auto-correlation, highlighting a strong hierarchy of the predictors in our study area. Climatic variables – mean annual precipitation and accumulated water deficit – emerged as the most important variables determining tree cover^14^. This result is not a surprise since it has been heavily demonstrated that climate, especially rainfall, constitutes the primary determinant for tree cover in Africa^5^,^11^,^42^. Indeed, water availability constrains the distribution of forest and savanna globally^2^,^6^,^43^,^44^, and within the savanna biome it drives differences in growth rates between trees and grasses and hence determines vegetation structure^45^.

Anthropogenic variables, specifically agriculture, also mattered in determining tree cover. Pastoralism in savannas is a major driver of vegetation structure^14^, but rarely, if ever, taken into account in global analyses^4^,^6^. Modifications of agricultural practices can lead to drastic changes in vegetation structure. For example, pastoralism in Central African savannas has long been only located in Sahelian areas, which is noticeable in our results where *grassland/bushland* and *sparsely wooded savanna* classes support the highest density of livestock (Fig. 3c). However, Sahelian herders from both West and Central African areas recently (~1960 s) migrated southward in mesic savannas where historically farmers dominated the agricultural landscape^16^,^32^. This situation has led to important conflicts between herders and farmers^32^,^46^, and to changes in vegetation structure. For example, the modifications in fire regime due to change in agricultural practices (from large fires for hunting to small and early fires for cattle ranching) associated with overgrazing resulted in bush encroachment^16^. Pasture areas became less productive, and herders migrated more southward^32^. Pastoralism is not the only agricultural practice that directly or indirectly impact tree cover. Traditional farmers use slash-and-burn to clear lands and manage fields and communal pastures^16^, and conversion to cropland obviously reduces tree cover^47^.

Fire also has negative impacts on intermediate tree cover^11^. Fire is highly related to agricultural activities and hunting, meanwhile recent changes in practices have reduced both the extent and intensity of fires^16^. These smaller fires, as well as fires in high-woody cover areas (such as for the *wooded and woodland savannas*), are difficult to detect using remote sensing products^48^,^49^. This may explain why fire is not playing a more important role in our model. Ultimately, edaphic variables were significant^2^, but less predictive of tree cover; soil organic carbon concentration was the most predictive soil variable (Fig. S2), which may reflect vegetation impacts on soil carbon, and not vice versa^50^.

When considering vegetation structural classes, the climatic variables appear also to be the main drivers of their geographical distribution (Fig. 3a,b), but the non-climatic drivers of vegetation classes shifts were very different from one class to another. First, the *wooded and woodland savannas* and the *forest* class clearly differ regarding their fire activity: the former features very high fire frequency, while the latter exhibits no fire (Fig. 3e). It has been previously proposed that these two vegetation types represent alternative stable states, maintained by a fire feedback^3^,^4^,^6^. As tree cover increases, beyond some point, the grass layer availability for fuelling fire and its flammability decrease, promoting the increase of tree densities. Conversely, if tree cover falls below a critical density, the light release to the ground increases promoting fuel flammability and availability, and thus the occurrence of fires that could kill tree seedlings^51^ and induce a change toward a more open landscape (*wooded and woodland savannas*).

Particular mechanisms that imply a transition from grassland to savanna in semi-arid ecosystems can be considered to understand transitions between the two most open vegetation classes. On one hand, a strong Allee effect in grasslands and bushlands, i.e. low shrub density limiting recruitment, can be strong enough to keep the system trapped in this state^52^,^53^. On the other hand, biotic stochasticity and spatial variability of disturbance regimes – such as selective herbivory, very local fires or rainfall events – can produce favourable conditions for tree recruitment^54^,^55^,^56^. Additionally, while some fires are detected in the *sparsely wooded savannas* class, very few fires are detected in the *grassland/bushland* ones (Fig. 3e) suggesting a change in disturbance regime from one class to the other due to differences in fuel availability.

The *sparsely wooded savannas* and the *wooded and woodland savannas* represent ecosystems that differ structurally from each other, potentially sustained by different processes and mechanisms^13^. Accordingly, soil characteristics are different between these two classes, especially cation exchange capacity, organic carbon and silt content (Fig. 3d–f–h). There might be a combination of soil properties and water availability limiting tree cover^57^. However, more information is needed to understand if this relationship is deterministic or results from feedbacks. Interestingly, the recorded fire frequency appears to be the same between the two savanna classes (Fig. 3e), while fire is almost unrecorded in the other classes. That might mean there is no relationship between fire frequency and tree cover at intermediate values because tree species in both savanna classes are adapted to similar level of disturbance intensities^58^. In this case, differences in traditional agricultural practices are the main driver of vegetation structure (Fig. 3c, pasture density). Alternatively it may be due to a limitation in the L3JRC product in recording burned area in zones of high tree cover^49^.

Our simulations of future tree cover highlighted differences between RCP scenarios. First, RCPs 2.6 and 8.5 resulted in similar outputs with increasing cropland areas as the main driver of vegetation change^14^. Interestingly, these are the two extreme scenarios in terms of CO_2_ emission and radiative forcing^18^, with RCP 2.6 achieving a drastic reduction in both because of a major increase in croplands for biofuel production^59^. Under RCP 8.5 the demand for agricultural land dedicated to cash crops would increase^59^. The consequences of tree cover decrease in forest areas are straightforward, with expected decrease in carbon storage and sequestration^60^ and important biodiversity loss due to agricultural conversion^26^,^61^. But, on the other hand, increasing areas for biofuel production in savannas also may have major issues. First, the consequences in term of carbon storage and biodiversity would be the same^62^. But also, allocating lands for biofuel and cash crop productions can create tensions for the availability of lands for local farmers and can raise food security issues^63^,^64^. Mesic savannas in Central Africa are considered as having a high cultivation potential^62^,^64^, but the tensions between herders and farmers have never been so high^32^. One can easily imagine how increasing areas for cash crops and biofuel production in the region may exacerbate the tensions that already exist for land availability^46^.

Conversely for RCPs 4.5 and 6.0, climate change was the major driver of tree cover change and vegetation shifts, with tree cover increase with increasing annual rainfall in Sahelian areas and tree cover decrease with increasing accumulated water deficit close to the Congo basin. This result agrees with other studies highlighting the role of seasonality in the future of forest and savanna in the tropics^20^,^65^. However, our simulations showed that land-use modifications through decreasing agricultural areas were able to counterbalance the consequence of increasing water deficit in forest areas^40^. This is a result of increasing the effort of reforestation policies, especially under RCP 4.5, as a way to mitigate climate change^59^.

Increasing annual rainfall resulted in an increase in tree cover in Sahelian area in our simulations. Other authors also noted the ‘greening’ and ongoing woody encroachment^66^,^67^ in this area as a result of precipitation change^68^. Woody encroachment may need to be strongly monitored because of its potential negative impacts on faunal and floral diversity^69^,^70^, and on the quality of grazing lawns for herds and cattle^16^. It is also important to note that the decrease in agricultural areas in savanna directly bordering forest resulted in increasing tree cover, with some areas of the *wooded and woodland savanna* class shifting to *forest*. These savannas are particularly sensitive to land-use change^14^. First they are located in a climate range where forest is possible^3^, such that changing the balance in disturbance can trigger a transition to forest. But also, because these savanna areas, which persisted for thousands of years, are increasingly targeted for afforestation policies^71^,^72^.

Finally, we must consider that future global change may result in responses beyond what we can predict on the basis of modern-day correlations. To produce these simulations of future tree cover, we used a Random Forest algorithm, such that our model as all other machine-learning algorithms generates only correlative relationships between ecological patterns and predictor variables^65^. As a consequence, our model uses the amplitude of tree cover response to the predictors constrained by the training data, i.e. the currently observed data, to simulate future responses.

We did not consider CO_2_ in our model, because up to now, little is known on how trees and grasses will respond to it, and more experimental work is needed to account for CO_2_ effects in predictive models. Similarly, we could not consider the direct effects of increasing temperature on vegetation since the present extant range of variability does not include projected future temperatures in the tropics^59^. We, however, took into account the effects of temperature changes on evapotranspiration and thus on water availability, since it is likely that temperature will mainly influence vegetation through water stress^44^,^73^,^74^. But, the high uncertainty in future climate projections that are related to GCM biases^75^ and that are known to be high in Africa^27^ constitute another source of uncertainty.

No projections on fire regime are available yet such that we decided not take into account fire in our projections of tree cover. Even if at a global scale fire is known to play a role in the distribution of savanna and forest by maintaining a feedback on vegetation structure^3^, we showed that there was no difference in fire frequency between the two savanna classes with the highest tree cover. Consequently, future changes in fire regimes may trigger the transition from savanna to forest, especially because fire suppression policies are widespread^18^, but may be less crucial for the future of savanna tree cover in comparison to other land-use changes.

## Conclusion

Here, we studied the determinants of tree cover in Central Africa, in an area where population density and conversion to intensive agriculture is low, and where traditional agricultural practices dominate. We showed that climate, and more particularly annual rainfall and accumulated water deficit, was the main driver of both tree cover and vegetation classes distribution. Agricultural practices, especially pastoralism, were also important in determining tree cover. We simulated future tree cover using a correlative model and by accounting for predicted changes in both climate and agricultural practices. We showed that, for some scenarios, land-use could result in tree cover changes of higher amplitude for 2070 than climate only, and for other it can counterbalance the effect of climate change alone. We thus emphasize that to provide more realistic projections of future vegetation and tree cover, it is essential to consider both climate change and direct human impact^14^.

Additionally, our simulations showed that for RCP 2.6, aggressive emissions reduction resulted in forest fragmentation and drastic tree cover decrease in the most wooded savanna. In this context, choosing to mitigate climate change can result in vegetation and biodiversity erosion. It thus raises the question of the way global communities are going to balance their climate change mitigation policies and priorities.

Finally, as statistical approaches should be supplemented with mechanistic approaches, more investigations are needed on climate-human-vegetation interactions. Developing such theoretical framework that includes human land-use may increase our understanding about future impacts on vegetation and could help make decisions about conservation priorities and management planning.

## Methods

### Study location and variables

#### Tree cover

We analysed tree cover distribution on a study area defined between latitude 0°N and 14°N, and longitude 13°E and 31°E (Fig. 1a,b). This area shows a clear vegetation gradient from tropical forest to open grasslands in Central Africa. The region is characterized by low population density^30^ and is still subject to very localized intensive land conversion^31^, with traditional agricultural practices^32^. Tree cover percentage was computed from the MOD44B Collection 3 product^34^ for the year 2000 and aggregated at 5 × 5 km resolution. It comprises the canopy cover percentage derived from Moderate Resolution Imaging Spectroradiometer (MODIS) satellite measurement of canopy reflectance.

To relate tree cover, the response variable of our study, with ecological vegetation classes, we used the frequency distribution of tree cover in the study area. We calculated the probability density function of tree cover in the study area to identify the major vegetation classes. We used finite normal mixture modelling to estimate the number of modes of the tree cover frequency distribution. This technique fits several frequency distributions to the data. We used the R package mclust (R version 3.1.3, mclust version 5.0.0) that uses an expectation-maximization (EM) procedure to find the best fit for several normal distributions. The Bayesian Information Criterion (BIC) was used to define the most optimized number of classes.

#### Potential explanatory variables

The vegetation structure in Africa is likely a result of interactions between climate, soil biochemistry, fire, herbivory, and human activities^2^. Here we used two climatic variables: the mean annual precipitation (MAP) derived from the 30-arcsec WorldClim Version 1.4 datasets^76^, and the yearly accumulated water deficit (AWD). Monthly average potential evapotranspiration (PET) were computed following the Hargreaves 1985 method^77^ and using mean monthly temperature and monthly range temperature from WorldClim Version 1.4^76^. This method has been tested in Africa and South America with very good results^78^. The AWD was then computed as following:


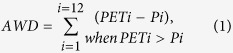


with *PETi* the potential evapotranspiration for month *i*, and *Pi* the precipitation for month *i*. Thus, here the accumulated water deficit of the dry season represents the best variable to capture water availability through precipitation seasonality and the effect of temperature in potential evapotranspiration.

The ISRIC World Soil Information team in collaboration with the AFSIS project (African Soil Information System) have produced predictions of soil properties at 1 km resolution for Africa using about 12,000 soil profile data^79^. The ISRIC soil properties estimates were used for six soil characteristics that can be potential determinants of savanna structure^2^,^5^,^11^: percent of sand, clay and silt, organic carbon content, pH (~phosphorus availability) and the cation exchange capacity (~fertility) in 100-cm of soil depth (data averaged). We used the monthly L3JRC burnt area product to derive an estimate of fire frequency^48^. For this analysis, monthly data layers from 2000 to 2007 were combined to calculate the total number of times individual pixels burned over the time period. We used population density data for the year 2000^80^; due to important differences in population densities across the study area, the data were log-transformed. Land-use data (cropland and pasture) were derived from the Harmonized Global Land Use for years 1700–2100 version 1^40^, available for the entire globe at 0.5-degree spatial resolution. Historical data (1700–2005) are based on HYDE 3.1; we averaged the density of cropland and pasture per pixel for the years 2000 to 2005.

In Africa, herbivores can represent an important disturbance^11^,^81^ thus playing a role in determining tree cover. Nevertheless, data availability on herbivore abundance is sparse and unreliable so we could not include herbivore abundance as a predictor.

All the data were re-projected in WGS-84 at a resolution of 5 km using a nearest neighbour procedure. After creating a regular grid of 142,993 points, average percent tree cover, fire frequency, climate, soil and land use data were extracted to perform statistical analysis.

### Modelling framework

Random Forest models use a classification or regression tree approach that recursively partition predictor variables. The algorithm creates multiple bootstrapped regression trees without pruning and averages the outputs; each tree is grown using a randomized subset of predictors^82^. These models are very effective in reducing variance and error in high dimensional data sets by taking an ensemble of unpruned trees. Moreover, growing large numbers of trees does not overfit data and random predictor selection keeps bias low, providing good models for prediction^35^. Several metrics are available to help interpreting these models. Variable importance can be evaluated based on how much worse the prediction would be if the data for that predictor were permuted randomly; it is thus possible to rank predictors based on their relative importance^82^. This type of models has been previously used to predict the impact of climate change on vegetation and biome distribution with good results^14^,^41^.

In order to avoid collinearity between soil variables, Pearson correlation matrix was computed for potential variables and only those with correlation coefficients with any other lesser than 0.7 were considered in the analysis (Table 1). We randomly selected seventy percent of the dataset to calibrate the model, and used the remaining 30% for validation. We also considered the spatial autocorrelation of the model’s residuals and applied Moran’s I, an index of spatial autocorrelation ranging from −1 to 1, where a positive value indicates a positive spatial autocorrelation, and vice et versa, and where values close to zero indicate no spatial autocorrelation^83^. Due to computational limitations, we assessed Moran’s *I* in a randomized subset of 10,000 pixels of the training data^84^.

All data were extracted and analyzed in R 3.1.3^85^, using libraries “raster”, “randomForest” and “ape”.

### Global changes scenarios and model projections

Representative Concentration Pathways (RCPs) represent the trajectory for greenhouse gas and radiative forcing reached by the year 2100^36^. RCPs are independent pathways produced by four individual modelling groups (Integrated Assessment Model, IAM): one high pathway for which radiative forcing reaches > 8.5 W.m-2 (~1370 ppm CO_2_ eq) by 2100 and continues to rise for some amount of time (MESSAGE); two intermediate “stabilization pathways” in which radiative forcing is stabilized at approximately 6 W.m-2 (~850 ppm CO_2_ eq; AIM) and 4.5 W.m-2 (~650 ppm CO_2_ eq; GCAM) after 2100; and one pathway where radiative forcing peaks at 2.6 W.m-2 (~490 ppm CO_2_ eq; IMAGE) before 2100 and then declines. Any differences between the pathways can be attributed in part to differences between models and scenario assumptions (scientific, economic, and technological). For example, the use of oil stays constant in most scenarios, but declines in the RCP 2.6, as a result of depletion and climate policy^59^. Moreover, the use of non-fossil fuels is expected to increase in all scenarios, especially using renewable resources such as wind and solar, bio-energy and nuclear power. Thus, a crucial element of RCP 2.6 is the use of bio-energy, carbon capture and storage technologies, which results in negative emissions^59^. The counterpart of this decrease nevertheless is a large increase in croplands dedicated to biofuel production^86^. For this scenario, pasture stays constant in our area (Fig. S4) but globally increases mildly. Meanwhile, RCP 4.5 projects a radical change in global land-use because in that scenario carbon storage from vegetation is valued as part of global climate policy^59^. Cropland and pasture, for this scenario, decrease as a combined result of reforestation programs, yield improvement, intensification and dietary changes^86^. RCP 6.0 assumes an increase in cropland, especially in urban areas, due to population and economic growth, but a decline in pasture as the result of a shift from extensive to more intensive animal husbandry^86^. Finally, the RCP 8.5 is also expected to increase croplands and pasture lands as a result of the large increase in global population^86^. Each RCP achieves its radiative forcing trajectories by simulating diverse land-use, socio-economic and policy scenarios, such that the intensity of land-use change does not monotonically increase with RCP radiative forcing^59^.

Future climate projections for 2070 (average 2061–2080) were taken from all the general circulation models (GCMs) for the four RCPs; the GCM outputs are available downscaled and calibrated against Worldclim 1.4 as baseline climate^87^, using absolute change for temperature and relative change for precipitation. Because we used an ensemble of all GCMs available, we derived consensus projections of tree cover for each RCP using principal components analysis. As described in Heubes *et al*.^27^, tree cover predicted for each GCM for a given RCP is weighted according to the first PCA axis loadings. Future land-use projections for the year 2070 were averaged from 2061 to 2080 for the four RCPs^40^ to be consistent with the climate data. Finally, human population density data were derived from IPCC SRES projections A1B^80^ for the year 2100, which forecasts an increase in population density followed by a stabilization and then a decline. The other two scenarios, A2 and B2, have similar projections for Africa.

To distinguish between climate vs. land-use change effects on tree cover, we simulated future tree cover with the Random Forest model we calibrated and using projected climate change (MAP and AWD), land-use change (pasture, cropland and population densities), and both^14^. We kept fire and soil constant in our simulations because standard future fire projections are not yet available, and changing soil variables would require building a more complex model taking into account feedbacks between soil and vegetation, which is not possible with a statistical model.

## Additional Information

**How to cite this article**: Aleman, J. C. *et al*. Tree cover in Central Africa: determinants and sensitivity under contrasted scenarios of global change. *Sci. Rep.* **7**, 41393; doi: 10.1038/srep41393 (2017).

**Publisher's note:** Springer Nature remains neutral with regard to jurisdictional claims in published maps and institutional affiliations.

## Supplementary Material

Supplementary Figures

## Figures and Tables

**Table 1:** Available and selected variables used in this study and for simulations of future tree cover.

Variable	Unit	Range	Selected in model	Used for future prediction
Tree cover	%	[0; 100] Mean = 36.8	✓	
Mean annual precipitation (MAP)	mm/year	[216; 2296]Mean = 1205.8	✓	✓
Accumulated water deficit (AWD)	mm/year	[0; 1311] Mean = 542.7	✓	✓
Sand	%	[6.5; 82.9] Mean = 47.9	✓	
Clay	%	[7.9; 70.7] Mean = 36.3		
Silt	%	[2.8; 52.9] Mean = 15.8	✓	
Organic carbon concentration	g/kg	[1.3; 65.3] Mean = 6.9	✓	
pH		[3.7; 8.8] Mean = 5.9		
Cation exchange capacity (CEC)	cmol/kg	[1.5; 209.6] Mean = 12.2	✓	
Fire frequency	Nb of fire per year	[0; 1] Mean = 0.12	✓	
Cropland	% of the pixel	[0; 0.6] Mean = 0.07	✓	✓
Pasture	% of the pixel	[0; 0.9] Mean = 0.3	✓	✓
Population density	Number of inhabitants per pixel (log-transformed)	[0; 11] Mean = 6.4	✓	✓
