# Supplementary material for: Tree cover in Central Africa: determinants and sensitivity under contrasted scenarios of global change
Source: Sci Rep. 2017 Jan 30;7:41393. doi: 10.1038/srep41393 (PMC5278362; doi:10.1038/srep41393)
Supplement: Supplementary Figures [file srep41393-s1.pdf]

Supplementary information for

Tree cover in Central Africa: determinants and sensitivity under contrasted scenarios of global change

Julie C. Aleman[1, 2, 3, *], Olivier Blarquez[4], Sylvie Gourlet-Fleury[3], Laurent Bremond[2] and Charly Favier[2]

[1] Ecology and Evolutionary Biology, Yale University, New Haven, USA.
[2] Institut des Sciences de l'Evolution de Montpellier, Université de Montpellier, CNRS, IRD, EPHE, Montpellier, France.
[3] UR Biens et services des écosystèmes forestiers tropicaux (CIRAD), Montpellier, France.
[4] Département de Géographie, Université de Montréal, Montréal, Québec, Canada.

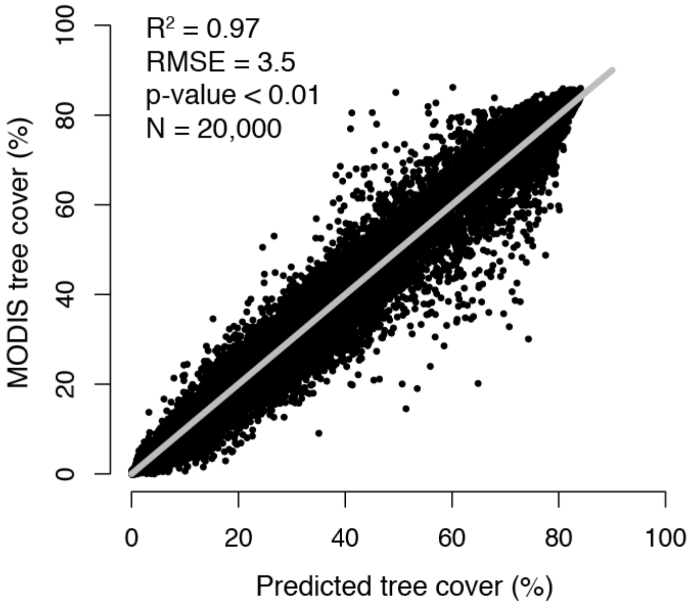

Fig. S1 – Predicted tree cover vs. MODIS tree cover for 20,000 points of the validation dataset.

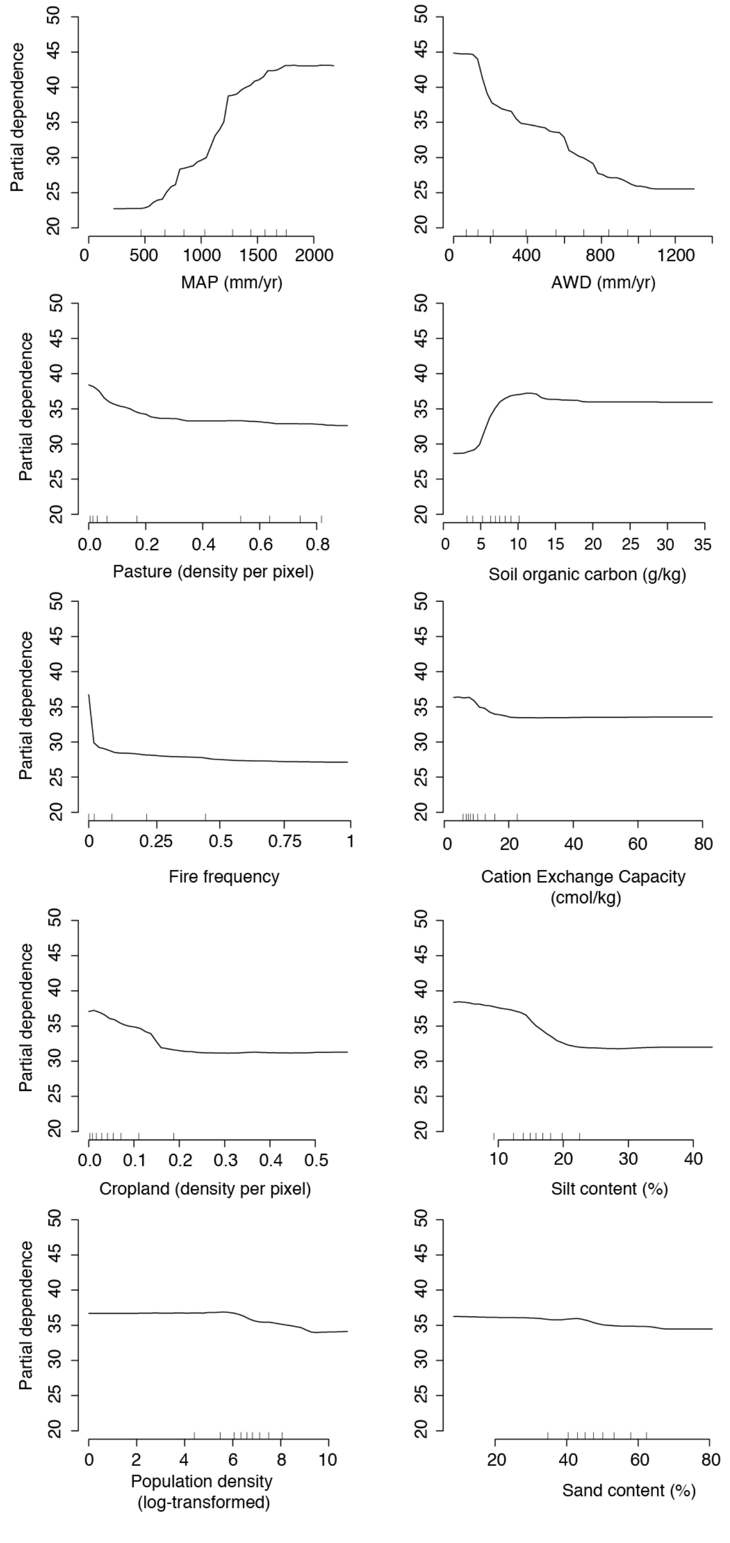

Fig. S2 – Partial dependence of the variables to the tree cover as predicted by the model.

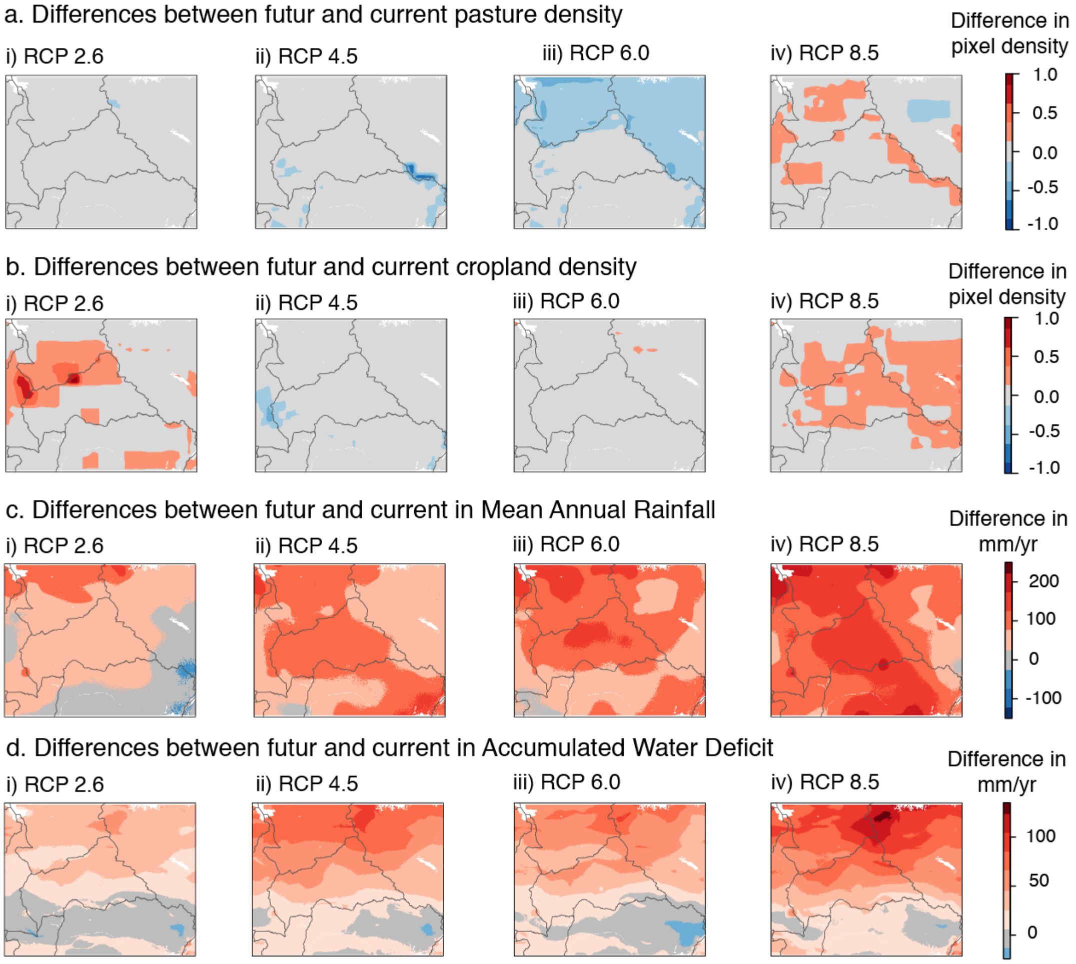

Fig. S3 – Major changes in climatic and anthropogenic predictors for 2070 regarding the four RCPs. The maps were generated using R version 3.1.3 [1].

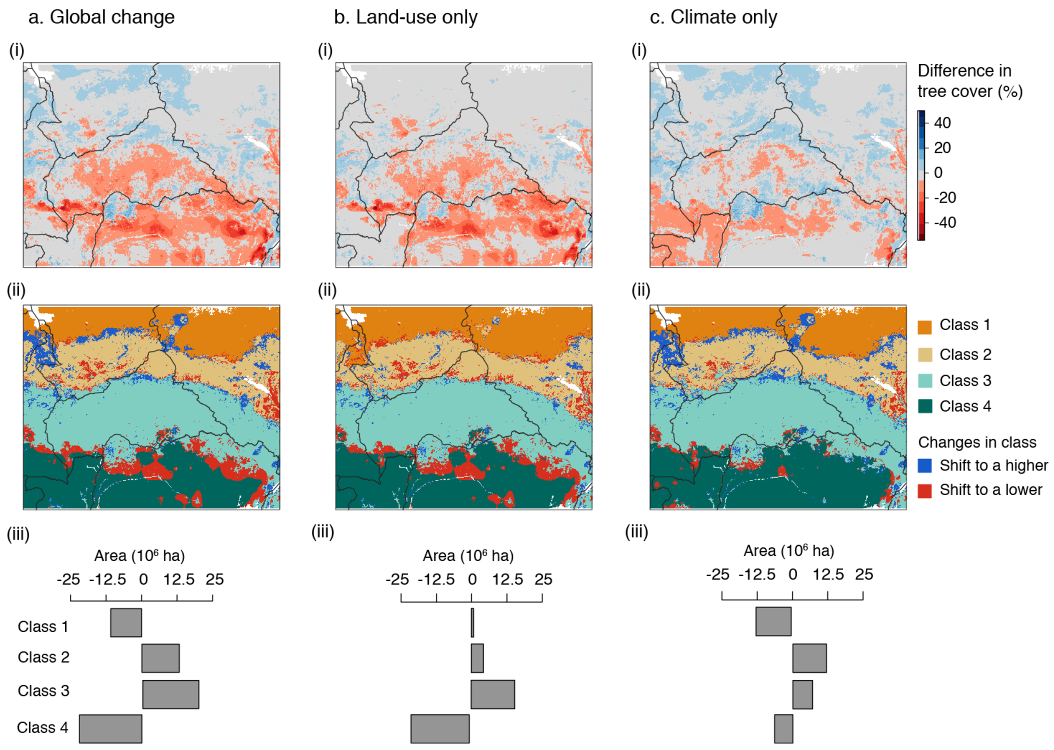

Fig. S4 | Changes in tree cover and classes' spatial distribution for RCP 8.5. The first panel represents differences in tree cover between simulated future and current tree cover values (i), the second panel represents the spatial distribution of the four vegetation classes and their shifts for 2070 (ii), and the third panel represents the changes in area occupied by the four vegetation classes in 2070 (iii) for the global change (a), land-use change only (b) and climate change only (c). The maps were generated using R version 3.1.3 [1].

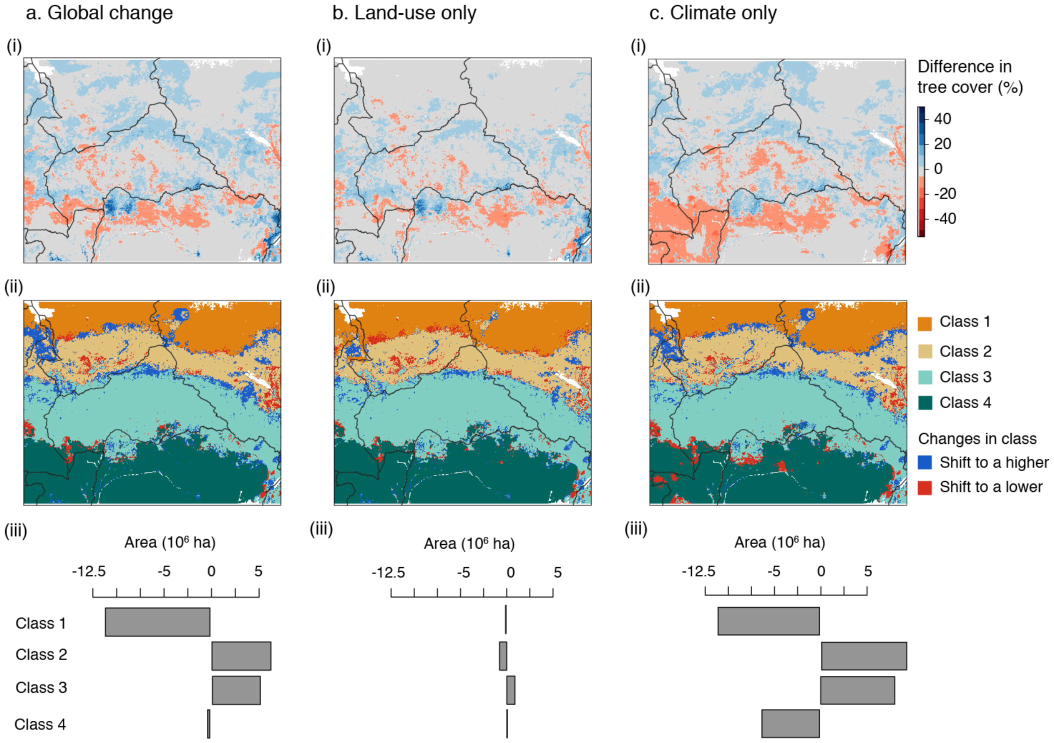

Fig. S5 | Changes in tree cover and classes' spatial distribution for RCP 6.0. The first panel represents differences in tree cover between simulated future and current tree cover values (i), the second panel represents the spatial distribution of the four vegetation classes and their shifts for 2070 (ii), and the third panel represents the changes in area occupied by the four vegetation classes in 2070 (iii) for the global change (a), land-use change only (b) and climate change only (c). The maps were generated using R version 3.1.3 [1].

1 R: A language and environment for statistical computing (R Foundation for Statistical Computing, Vienna, Austria., 2015).
